# Integrating phylogenomic and morphological evidence to solve the taxonomy of *Phoebe legendrei* (Lauraceae) and closely related species

**DOI:** 10.1186/s12870-025-07929-1

**Published:** 2025-12-13

**Authors:** Chengyan Shao, Zhi Yang, Yuyu Ye, Jiangbin Lin, Jianyong Lin, Rihong Jiang, David Kay Ferguson, Bing Liu, Yong Yang

**Affiliations:** 1 Co-Innovation Center for Sustainable Forestry in Southern China, College of Life Sciences, Nanjing Forestry University, 159 Longpan Rd, Nanjing, 210037 China; 2 Sichuan Panzhihua Cycad National Nature Reserve Protection Center, Panzhihua, 617000 China; 3 Key Laboratory of Central South Fast-growing Timber Cultivation of Forestry Ministry of China, Guangxi Key Laboratory of Superior Timber Trees Resource Cultivation, Guangxi Forestry Research Institute, Nanning, 530028 China; 4 Department of Paleontology, University of Vienna, Vienna, Austria; 5 State Key Laboratory of Plant Diversity and Specialty Crops, Institute of Botany, Chinese Academy of Sciences, Beijing, 100093 China; 6 Sino-Africa Joint Research Center, Chinese Academy of Sciences, Wuhan, 430074 China

**Keywords:** Lauraceae, *Phoebe panzhihuaensis*, Plastome, Phylogeny, Integrative taxonomy

## Abstract

**Background:**

The genus *Phoebe*, belonging to the tribe Perseeae of the Lauraceae, is well known for its important economic value and the need for its conservation. However, the taxonomic delimitation of species remains poorly resolved due to complicated morphological variation in *Phoebe*, thus hindering conservation efforts and resource utilization. Here we sequenced 18 samples of the genus, reconstructed new plastome phylogenomic trees of *Phoebe* from East Asia with special emphasis on *Phoebe legendrei* and its closely related species. We also conducted comparative analyses of both plastomes and morphology between *P. legendrei* and its closely related species, and examined the protologue and the type collection of *P. legendrei*, and conducted a taxonomic treatment.

**Results:**

Our comparative genomic analysis confirmed that plastomes of the genus *Phoebe* are relatively conservative, but *P. legendrei* 94 differs from *P. legendrei* 143 in number of dispersed repeats, IR boundary, and highly variable regions. The newly reconstructed phylogenomic trees are robustly supported, with *Phoebe* species divided into three major clades. *Phoebe crassipedicella* is sister to *P. kwangsiensis*, but the eight samples of *P. legendrei* formed two separate subclades (represented by *P. legendrei* 94 differs from *P. legendrei* 143, respectively). We conducted a morphological study and revealed that the two subclades differed in perianth lobe size, fruiting pedicel length, and the clasping status of the persistent perianth lobes. We examined the protologue and morphology of the type collection of *P. legendrei*, and determined a new species, i.e., *Phoebe panzhihuaensis* sp. nov.

**Conclusions:**

In this study, we resolved the taxonomic delimitation within *P. legendrei* and its closely related species, and discovered a new species of *Phoebe* from southwestern China. This work contributes to evaluating species diversity within *Phoebe* and lays the foundation for the conservation and resource utilization of these species.

**Supplementary Information:**

The online version contains supplementary material available at 10.1186/s12870-025-07929-1.

## Introduction

The genus *Phoebe* Nees [1], belonging to the trib. Perseeae of the Lauraceae [2], contains ca. 100 species distributed in tropical and subtropical Asia [3]. There are ca. 30 species living in southern China [4]. Delimitation of the genus is supported by both morphological and molecular evidence. *Phoebe* differs from other genera in the trib. Perseeae in that the fruits are ellipsoidal or ovoid, and the perianth lobes are persistent and clasping the base of fruits [5, 6]. Phylogenetic studies have consistently indicated that the genus *Phoebe* is monophyletic [7–12].

*Phoebe* species have been used for different purposes, e.g. timber and medicine. In China, timber of *Phoebe* is considered to be excellent and widely utilized for construction, furniture and carvings, because of its unusual characteristics including remarkable durability, natural decay resistance, and aesthetic grain patterns [13]. In particular, gold-thread nanmu (*Phoebe* timber) was once the timber specifically used by the royal family during the Ming and Qing dynasties. As a result, it was considered as the royal wood. Moreover, the secondary metabolites of *Phoebe* species, such as alkaloids and terpenoids, exhibit pharmacological effects including antitumorigenic, antimalarial, and antibacterial properties [14]. However, due to habitat deterioration and over-exploitation [11, 15], approximately 50% of the native *Phoebe* species have become endangered [16], leading to several species being listed in the National Key Protected Wild Plant Species, e.g. *P. chekiangensis* C.B.Shang, *P. bournei* (Hemsl.) Yen C.Yang, *P. hui* W.C.Cheng ex Yen C.Yang and *P. zhennan* S.K.Lee et F.N.Wei [17]. Despite its economic and conservation significance, the taxonomy of *Phoebe* species is challenging, which limits their sustainable utilization and effective conservation.

The morphological differences among the *Phoebe* species are subtle, and distribution ranges of closely related species often overlap, which makes species delimitation problematic. *Phoebe chekiangensis* and *P. sheareri* (Hemsl.) Gamble exhibit overlapping distribution ranges in Zhejiang and adjacent provinces, their leaves are obovate, and branchlets, petioles and perianth lobes are pubescent [5]. They were once treated as a single species. It was not until Shang conducted comparative morphological studies that the two species were distinguished, with *P. chekiangensis* established as a new species [18]. This taxonomic conclusion has subsequently been supported by molecular evidence [11, 19]. *Phoebe zhennan* and *P. bournei* are widely distributed in central and southern China. Their morphological similarities have resulted in long-standing taxonomic debates [4, 5, 20]. Recently, Ding et al. [21] integrated morphological, genetic structure and phylogenetic evidence and concluded that *P. bournei* and *P. zhennan* should be divided into two separate species. Taxonomic delimitation has provided an important scientific basis for conservation and resource utilization of *Phoebe* plants.

Our morphological observations of both wild populations and herbarium specimens presented similar taxonomic challenges in *P. legendrei* Lecomte and *P. neurantha* Gamble. The widespread *P. neurantha* and narrow-ranged *P. legendrei* are similar in their lanceolate or oblanceolate leaves, pubescent branchlets, leaves, and petioles, ovoid fruits, lax persistent perianth lobes, leading to taxonomic confusion [5]. In addition, Wei et al. [22] noted the morphological similarity between *P. crassipedicella* S.Lee et F.N.Wei and *P. legendrei* when he described the former as a new species. It remains unclear if the two species are reciprocally monophyletic. It is therefore necessary to conduct an integrative taxonomic study to resolve the phylogenomic relationships and taxonomic delimitations of *P. legendrei* and similar species [23–27].

In order to examine the phylogenomic relationships and taxonomic delimitation of *P. legendrei* and similar species, we obtained and sequenced 18 samples representing seven species from herbarium specimens and field collections, performed a comparative study of plastomes between *P. legendrei* and its closely related species, reconstructed phylogenomic trees, and conducted a morphological study. By integrating morphological and molecular evidence, we clarified the phylogenomic relationships and species delimitation.

## Materials and methods

### Taxon sampling and field investigation

We obtained samples from multiple sources. We conducted field investigations and collected samples of *Phoebe* from Guangdong, Guangxi, Hainan, Hubei, Sichuan, and Yunnan, and obtained two herbarium samples from the Herbarium of the Guangxi Institute of Botany (IBK). We made additional collections and conducted morphological observations on the two populations of *P. legendrei* in Sichuan Panzhihua Cycad National Nature Reserve due to their diphyletic nature in the phylogenomic trees. In total, we obtained 18 samples representing seven *Phoebe* species for sequencing. Details of samples are provided in Table 1.

To reconstruct the phylogenomic trees of *Phoebe*, we also obtained six plastomes of *Phoebe* from Lin et al. [12] (Table 1) and downloaded 35 plastomes of *Phoebe* from NCBI (accessed 10 February 2025) (Table 2). In addition, to verify the monophyly of *Phoebe* and root the phylogenomic trees, we selected outgroups from the trib. Perseeae and three other tribes closely related to the trib. Perseeae [28]. The outgroups included *Machilus balansae* (Airy Shaw) F.N.Wei et S.C.Tang, *Persea americana* Mill., *Caryodaphnopsis malipoensis* Bing Liu et Y.Yang, *Litsea auriculata* S.S.Chien et W.C.Cheng, and *Ocotea tabacifolia* (Meisn.) Rohwer (Table 2).

**Table 1:** Voucher specimens of 24 samples in this study

Samples	Collection	Locality	Identification	Date	Herbarium	Accession
*Phoebe bournei* (Hemsl.) Yen C.Yang 1311	S.Z. Zhang SZBG-00011298	Shenzhen Fairy Lake Botanical Garden, Guangdong, China	S.Z. Zhang	27 May 2020	SZBG	PX297399
*Phoebe calcarea* S.Lee et F.N.Wei 2	J.Y. Lin and R.H. Jiang 220, 706,004	Jingxi City, Guangxi, China	R.H. Jiang	6 Jul. 2022	-	PX363577
*Phoebe jingxiensis* J.Y.Lin et R.H.Jiang 3	J.Y. Lin and R.H. Jiang 2, 009,010	Jingxi City, Guangxi, China	R.H. Jiang	14 Apr. 2020	GXFI	PX363578
*Phoebe jingxiensis* J.Y.Lin et R.H.Jiang 4	J.Y. Lin and R.H. Jiang 220, 705,018	Jingxi City, Guangxi, China	R.H. Jiang	5 Jul. 2022	-	PX363579
*Phoebe jingxiensis* J.Y.Lin et R.H.Jiang 5	J.Y. Lin and R.H. Jiang 220, 705,019	Jingxi City, Guangxi, China	R.H. Jiang	5 Jul. 2022	-	PX363580
*Phoebe jinpingensis* Bing Liu et al. 1477	B. Liu 1477	Jinping County, Yunnan, China	B. Liu	9 Oct. 2011	PE	PX297391
*Phoebe jinpingensis* Bing Liu et al. 2417	B. Liu et al. 2417	Jinping County, Yunnan, China	B. Liu	14 Sept. 2014	PE	PX297392
*Phoebe kwangsiensis* H.Liu 3007	Y.S. Huang, Y.B. Liao and R.C. Peng y0165	Mulun National Nature Reserve, Guangxi, China	F.N. Wei	1 May 2011	IBK	PX297393
*Phoebe kwangsiensis* H.Liu 9711	Y.G. Wei 90,106	Libo County, Guizhou, China	Y. Yang	28 Sept. 1990	IBK	PX297394
*Phoebe lanceolata* (Nees) Nees 2542	B. Liu, Y. Yang and T.W. Xiao 2542	Menghai County, Yunnan, China	B. Liu	28 Mar. 2015	PE	PX297400
*Phoebe legendrei* Lecomte 91	Y. Yang et al. PAN2-91	Panzhihua Cycad National Nature Reserve, Sichuan, China	Y. Yang	28 Jun. 2022	NF	PX297395
*Phoebe legendrei* Lecomte 92	Y. Yang et al. PAN2-92	Panzhihua Cycad National Nature Reserve, Sichuan, China	Y. Yang	28 Jun. 2022	NF	PX297396
*Phoebe legendrei* Lecomte 93	Y. Yang et al. PAN2-93	Panzhihua Cycad National Nature Reserve, Sichuan, China	Y. Yang	28 Jun. 2022	NF	PX297397
*Phoebe legendrei* Lecomte 94	Y. Yang et al. PAN2-94	Panzhihua Cycad National Nature Reserve, Sichuan, China	Y. Yang	28 Jun. 2022	NF	PX297398
*Phoebe legendrei* Lecomte 143	Y. Yang et al. PAN2-143	Panzhihua Cycad National Nature Reserve, Sichuan, China	Y. Yang	28 Jun. 2022	NF	PX297401
*Phoebe legendrei* Lecomte 144	Y. Yang et al. PAN2-144	Panzhihua Cycad National Nature Reserve, Sichuan, China	Y. Yang	28 Jun. 2022	NF	PX297402
*Phoebe legendrei* Lecomte 145	Y. Yang et al. PAN2-145	Panzhihua Cycad National Nature Reserve, Sichuan, China	Y. Yang	28 Jun. 2022	NF	PX297403
*Phoebe legendrei* Lecomte 147	Y. Yang et al. PAN2-147	Panzhihua Cycad National Nature Reserve, Sichuan, China	Y. Yang	28 Jun. 2022	NF	PX297404
*Phoebe nanmu* (Oliv.) Gamble 2222	B. Liu 2222	Wuhan Botanical Garden, Hubei, China	B. Liu	6 May 2014	PE	PX310284
*Phoebe nanmu* (Oliv.) Gamble 2227	B. Liu 2227	Wuhan Botanical Garden, Hubei, China	B. Liu	8 May 2014	PE	PX310285
*Phoebe puwenensis* W.C.Cheng 2643	B. Liu, Y. Yang and T.W. Xiao 2643	Cangyuan County, Yunnan, China	B. Liu	1 Apr. 2015	PE	PX297405
*Phoebe puwenensis* W.C.Cheng 2665	B. Liu, Y. Yang and T.W. Xiao 2665	Cangyuan County, Yunnan, China	B. Liu	1 Apr. 2015	PE	PX297406
*Phoebe yaiensis* S.K.Lee 1	J.Y. Lin and R.H. Jiang 2, 012,002	Sanya City, Hainan, China	R.H. Jiang	12 Dec. 2020	-	PX363581
*Phoebe yaiensis* S.K.Lee 2	J.Y. Lin and R.H. Jiang 2, 012,003	Sanya City, Hainan, China	R.H. Jiang	12 Dec. 2020	-	PX363582

**Table 2:** Accession numbers of plastomes obtained from NCBI in this study

Species	Accession	Species	Accession
*Phoebe calcarea* S.Lee et F.N.Wei	MZ433404	*Phoebe macrocarpa* C.Y.Wu	NC_058730
*Phoebe calcarea* S.Lee et F.N.Wei	NC_058725	*Phoebe neurantha* (Hemsl.) Gamble	MH394352
*Phoebe chekiangensis* C.B.Shang	MZ433405	*Phoebe neurantha* (Hemsl.) Gamble	MH394353
*Phoebe chekiangensis* C.B.Shang	NC_034925	*Phoebe neurantha* (Hemsl.) Gamble	MH394354
*Phoebe crassipedicella* S.Lee et F.N.Wei	MZ433406	*Phoebe neurantha* (Hemsl.) Gamble	MH394355
*Phoebe crassipedicella* S.Lee et F.N.Wei	NC_058726	*Phoebe neurantha* (Hemsl.) Gamble	NC_039620
*Phoebe bournei* (Hemsl.) Yen C.Yang	MT621604	*Phoebe neuranthoides* S.K.Lee et F.N.Wei	MZ433414
*Phoebe formosana* (Hayata) Hayata	MZ433407	*Phoebe neuranthoides* S.K.Lee et F.N.Wei	NC_058731
*Phoebe formosana* (Hayata) Hayata	NC_058727	*Phoebe sheareri* (Hemsl.) Gamble	KX437773
*Phoebe glaucophylla* H.W.Li	MZ433408	*Phoebe sheareri* (Hemsl.) Gamble	MT621573
*Phoebe glaucophylla* H.W.Li	NC_058728	*Phoebe sheareri* (Hemsl.) Gamble	NC_031191
*Phoebe hui* W.C.Cheng ex Yen C.Yang	MT621612	*Phoebe tavoyana* Hook.f.	MZ442607
*Phoebe hui* W.C.Cheng ex Yen C.Yang	OM022239	*Phoebe tavoyana* Hook.f.	NC_058829
*Phoebe hunanensis* Hand.-Mazz.	MT246867	*Phoebe zhennan* S.K.Lee et F.N.Wei	MF315089
*Phoebe hunanensis* Hand.-Mazz.	MZ433409	*Phoebe zhennan* S.K.Lee et F.N.Wei	NC_036143
*Phoebe hungmoensis* S.K.Lee	NC_079583	*Caryodaphnopsis malipoensis* Bing Liu et Y.Yang	NC_085767
*Phoebe hungmoensis* S.K.Lee	OQ468272	*Litsea auriculata* S.S.Chien et W.C.Cheng	MW355498
*Phoebe lanceolata* (Nees) Nees	MZ433411	*Machilus balansae* (Airy Shaw) F.N.Wei et S.C.Tang	NC_028074
*Phoebe lanceolata* (Nees) Nees	NC_058729	*Ocotea tabacifolia* (Meisn.) Rohwer	NC_061548
*Phoebe macrocarpa* C.Y.Wu	MZ433412	*Persea americana* Mill.	KX437771

### DNA extraction and genomic sequencing

This study employed different DNA extraction methods depending on the types of material. Total genomic DNAs were extracted from 16 silica-gel-dried samples using the GeneBetter Plant Genomic DNA Extraction Kit (Model: D115-100). For the two herbarium specimens, DNAs were extracted using the modified CTAB method (mCTAB) [29]. Key procedures including sample quality control, library construction, genome skimming, and assessment were performed by Novogene Co., Ltd. (Tianjin, China). The purity and integrity of the DNA samples were analyzed using agarose gel electrophoresis, while DNA concentration was precisely quantified using Qubit 2.0. Samples meeting the quality requirements were used for library construction. Genome skimming was performed on the Illumina NovaSeq 6000 platform, ensuring a minimum data output of 2 GB per sample. Finally, the quality of the raw sequence reads was assessed using FastQC, and ambiguous and low-quality reads filtered out.

### Genome assembly and annotation

Plastomes were assembled under Linux with GetOrganelle v1.7.5.0 [30], and assembly graphs were visualized in Bandage v0.8.1 [31]. Initial annotation was conducted with the web servers Geseq [32] and CPGAVAS2 [33], using *P. sheareri* (MT621573) as reference. Then annotations were manually curated in Geneious Prime 2023.2.1 [34]. Circular plastid genome maps of *P. legendrei* and *P. kwangsiensis* were drawn with OGDRAW v1.3.1 [35].

### Plastome genomic characterization

The plastome characteristics of *P. legendrei* and *P. kwangsiensis*, and their closely related species were analyzed, encompassing 11 sequences from 10 species (including two sequences from *P. legendrei*). The protein-coding genes (CDS) were extracted from the 11 plastomes using Geneious Prime 2023.2.1 for relative synonymous codon usage (RSCU) and selection pressure analysis [34]. To minimize errors, duplicate sequences were removed, and only CDS ≥ 300 bp with correct coding annotations were retained [36]. RSCU was calculated using MEGA 11.0.13 [37]. RSCU values > 1, = 1, and < 1 indicate high-frequency, neutral, and low-frequency codons, respectively [38, 39]. After removing duplicate CDS sequences, we used KaKs_Calculator 3.0 to calculate the non-synonymous substitution rate (Ka)/synonymous substitution rate (Ks) values, with *P. neurantha* (MH394352) being the reference sequence [11, 40]. The resulting Ka/Ks ratios were used to infer selective pressures on protein-coding genes, with values > 1 indicating positive selection, ≈ 1 suggesting neutral evolution, and < 1 reflecting purifying selection [41].

Simple sequence repeats (SSRs) were identified via the MISA web server [42], with repeat thresholds set to 10, 5, 4, 3, 3, and 3 for mono-, di-, tri-, tetra-, penta-, and hexanucleotide SSRs, respectively [43]. Dispersed repeats, including forward (F), reverse (R), complement (C) and palindromic (P), were detected using REPuter v2 [44], with minimum and maximum repeat lengths of 8 bp and 50 bp. All results were visualized using the R package ggplot2 3.5.2 [45].

### Comparative analyses of plastomes and identification of variable hotspots

IR boundary contraction and expansion among the 11 plastomes were compared and visualized with CPJSdraw v1.0.0 [46]. We used mVISTA [47] for the synteny analysis of 11 plastomes using *P. calcarea* as the reference, with default parameters and LAGAN and Shuffle-LAGAN mode. To identify highly variable regions in *Phoebe*, nucleotide diversity (Pi) was calculated in DnaSP v6.0 [48], with a window length set at 600 bp and a step size of 200 bp [49]. Variations were extracted using Geneious Prime 2023.2.1 [34].

### Phylogenomic analyses

We employed 64 plastomes for phylogenomic reconstruction, including 59 plastomes of *Phoebe* and five outgroup plastomes. Complete plastomes were aligned with MAFFT v7 [50] and automatically trimmed using trimAl 1.4.1 [51]. Phylogenomic reconstructions were conducted using both maximum likelihood (ML) and Bayesian inference (BI) approaches. Under the Bayesian Information Criterion (BIC), ModelFinder selected K3Pu + F + I + G4 as the best-fit substitution model [52]. ML trees were then inferred with IQ-TREE v2.3.6 [53], and branch support assessed via 5,000 ultrafast bootstrap (UFBoot) replicates [54]. For the BI tree, the best-fit model (GTR + F + I + G4) was selected under the BIC using PhyloSuite v1.2.2 and applied to the BI analysis in MrBayes 3.2.7 [52, 55]. Two independent Markov chain Monte Carlo (MCMC) runs were performed, each with two million generations, sampling every 1,000 generations, and diagnosing convergence every 5,000 generations. The resulting average standard deviation of split frequencies was 0.00440 (< 0.01), indicating that the runs had converged. Finally, the phylogenomic trees were visualized using the Interactive Tree of Life (iTOL) v6 [56].

### Morphological analyses

A total of 17 morphological characters were selected to conduct a morphological comparison between *P. legendrei* and closely related species. Morphological data were obtained from observations of herbarium specimens, published literature [12], *Flora of China*, and *Flora Reipublicae Popularis Sinicae*. These characters comprise seven quantitative characters, including length and width of leaves and fruits, number of leaf veins, and the length of petioles, inflorescences, infructescences, and fruit pedicels. Ten qualitative characters were assessed, such as the pubescence of branches, leaves, petioles and perianth lobes, shapes of leaves, perianth lobes and fruits, midvein prominence, adherence of persistent perianth lobes to fruit, as well as pedicel thickening [27]. Detailed morphological observations and photographs of *P. legendrei* were made under a Stereo Microscope (OLYMPUS SZX10). To delineate the morphological distinctions among *P. legendrei* 91–94, *P. legendrei*143, 144, 145, and 147, and *P. hui*, we performed principal component analyses (PCA) of 11 characters from 100 specimens from the Chinese Virtual Herbarium (CVH, https://www.cvh.ac.cn). To minimize measurement errors, mean values were used for the seven quantitative characters (leaf length, leaf width, number of lateral veins, petiole length, inflorescences length, infructescence length, and fruiting pedicel length). The four qualitative characters (leaf shape, perianth lobes shape, and state of persistent perianth lobes and fruiting pedicel) were coded as binary states (1 or 0). Two PCA analyses were conducted using the R package missMDA v 1.20 on two sets of characters: one with quantitative characters only, and the other combining both quantitative and qualitative characters [57]. The results were visualized using the R package ggplot2 3.5.2 [45].

## Results

### Characteristics of the 11 plastomes in the genus *Phoebe*

**Fig. 1:**
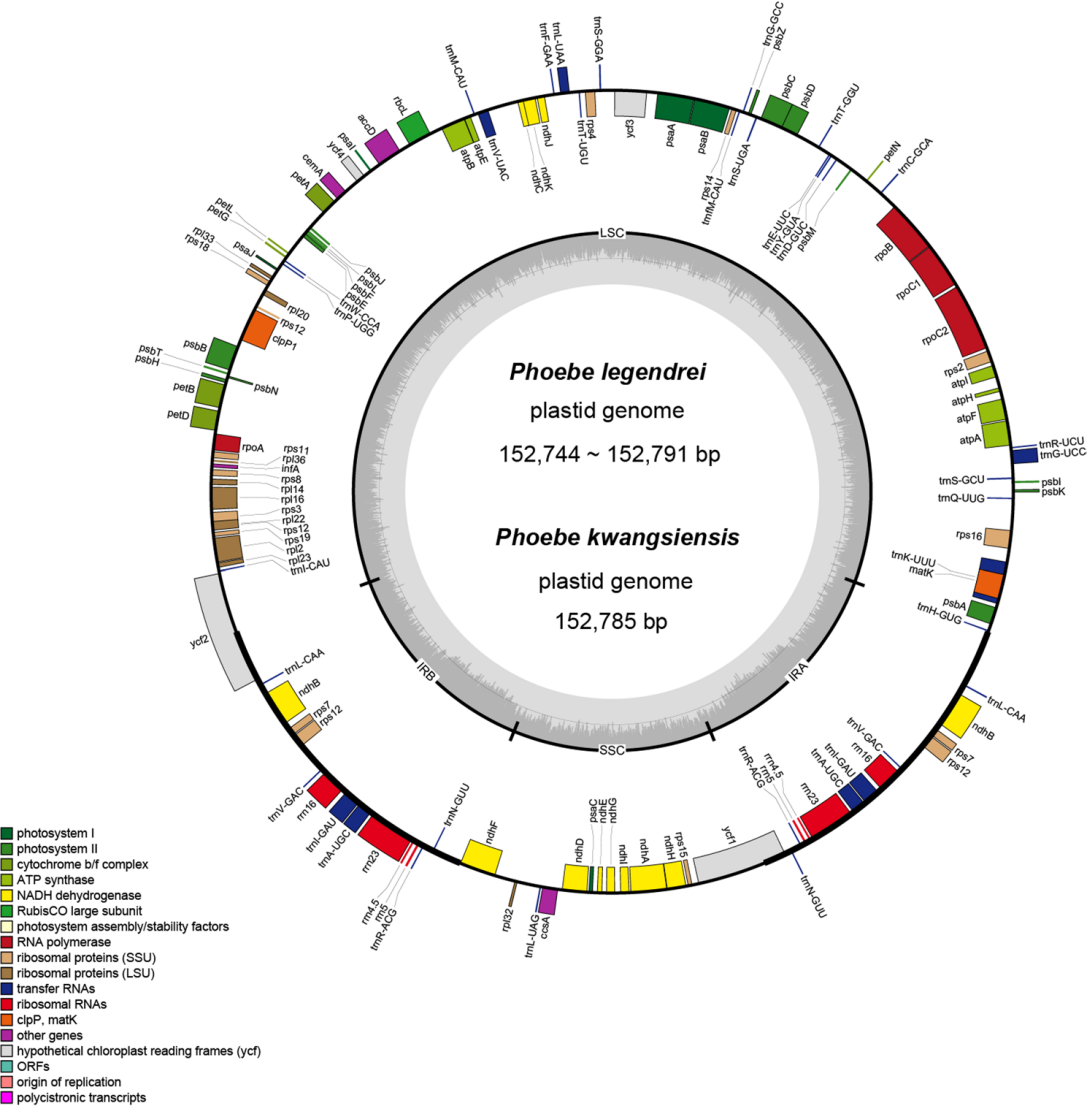
The plastome map of *P. legendrei* Lecomte and *P. kwangsiensis* H.Liu. In the map, genes on the outside are transcribed counterclockwise, and genes on the inside are transcribed clockwise. The dark gray area in the inner circle indicates the GC content, and the light gray area shows the AT content

Sixteen newly sequenced plastomes were successfully assembled to complete the circle (the plastomes of *P. nanmu* were assembled into linear). The plastid genome of the genus *Phoebe* is relatively conservative in structure, size, and gene composition. All plastomes of *Phoebe* share the typical quadripartite structure in which two inverted repeat regions (IRa and IRb) are separated by the large single copy region (LSC) and the small single copy region (SSC). The complete plastomes of *P. kwangsiensis*, *P. legendrei*, and closely related species ranged from 152,537 to 152,851 bp, while the total GC content was 39.1–39.2%. Detailed lengths and GC contents of each region are provided in Table S1. In total, we identified 126 genes, including 82 protein-coding genes, 36 tRNA genes, and 8 rRNA genes (Fig. 1, Table S1).

#### Codon usage bias analysis and Estimation of evolutionary rates

A total of 902 protein-coding genes (CDS) were extracted from the 11 plastomes. Based on specified criteria, 572 CDS were retained ultimately for RSCU analysis. There was almost no difference in the codon bias of the 11 sequences in the genus *Phoebe*. The relative synonymous codon usage (RSCU) values ranged from 0.33 to 1.83. The highest RSCU values (> 1.7) belonged to AGA (1.83), GCU (1.79) and UUA (1.75), which encode Arginine (Arg), Alanine (Ala) and Leucine (Leu), respectively. The lowest RSCU values (< 0.4) were observed for GCG (0.38), CGC (0.35) and AGC (0.33), which encode the Alanine (Ala), Arginine (Arg) and Serine (Ser), respectively. Thirty of the 59 codons had RSCU values > 1, and all of these ended in A or U with the exception of UCC (encoding Serine) (Fig. 3). This suggested that the 11 sequences of the genus *Phoebe* had a preference for A/U-ending codons.

Across the 10 plastomes, the 79 CDS genes exhibited Ka/Ks values ranging from 0 to 1.89766 (Table S2). Among all estimates, only 39 values were greater than 0, while 95.06% were 0. Three genes showed signals of positive selection (Ka/Ks > 1): the *ycf1* gene of *P. yaiensis* 1 (1.89766), the *ycf2* gene of *P. puwenensis* 2643 (1.48063), and the *ycf1* gene of *P. lanceolata* 2542 (1.25037). The *ycf1* gene of *P. tavoyana* NC_058829 had a value close to 1. The remaining 35 Ka/Ks values were all less than 1, consistent with the widespread purifying selection.

**Fig. 2:**
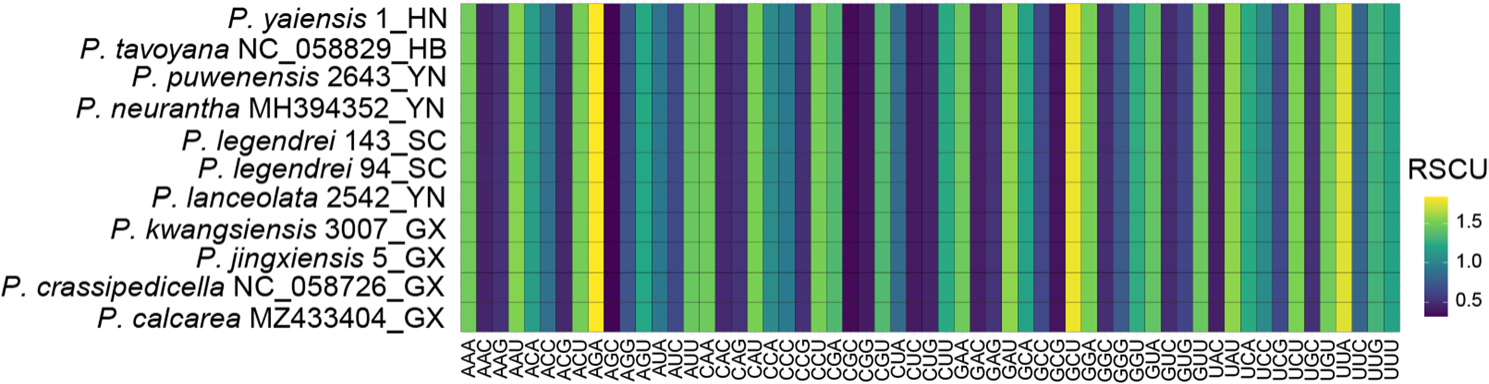
Relative synonymous codon usage (RSCU) in 11 plastomes of the genus *Phoebe.*The ending two capitalized letters of the sequence name denote the initials of the collection province

#### Simple sequence and dispersed repeats

**Fig. 3:**
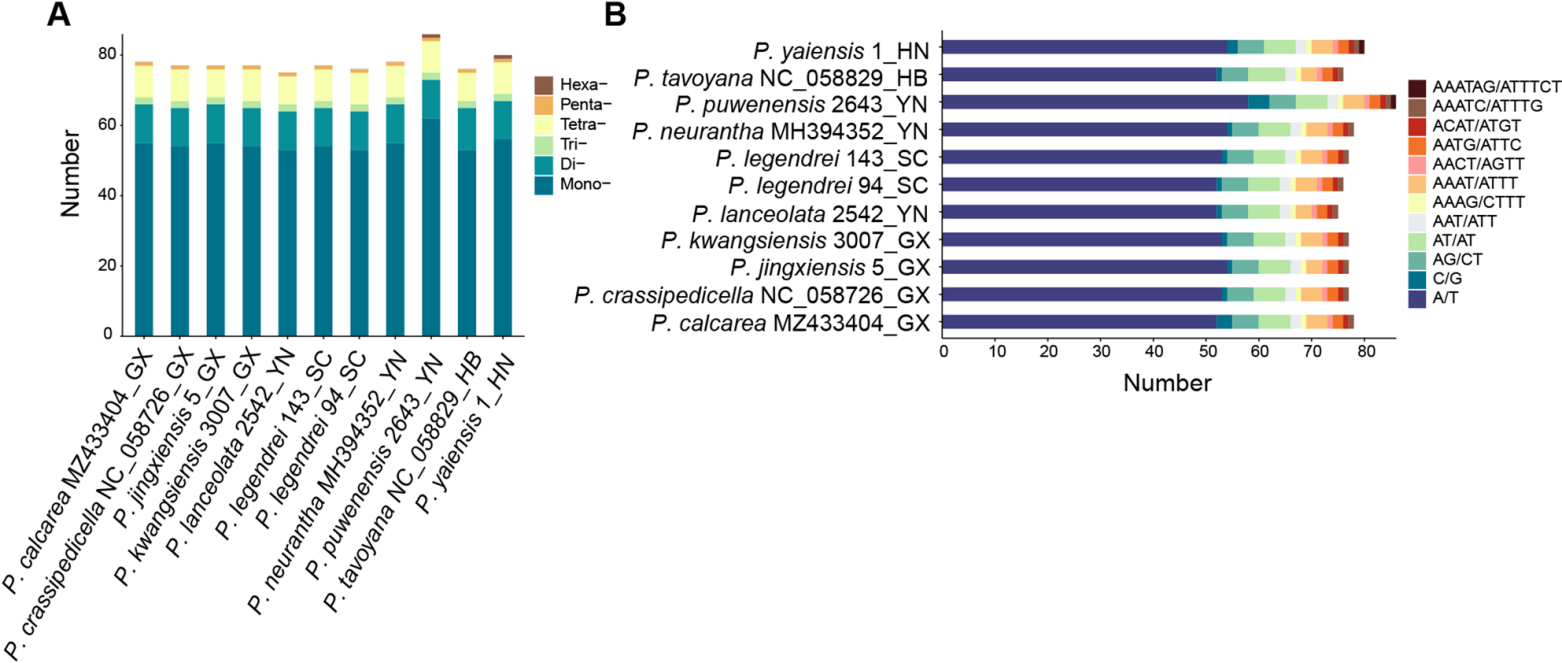
Statistics of SSRs in 11 plastomes. **A**: Number of the six SSR types; **B**: Number of SSR motifs in different repeat class types. The ending two capitalized letters of the sequence name denote the initials of the collection province

The number of simple sequence repeats (SSRs) in the 11 plastomes was 75 (*P. puwenensis* 2643) − 86 (*P. lanceolata* 2542) (Fig. 3A). Mononucleotide repeats were the most abundant (53–62), accounting for 69.7–72.1% of all SSRs. These SSRs were made up of 12 distinct motif types. Trinucleotide repeats exhibited the lowest diversity, represented solely by AAT/ATT, whereas tetranucleotide repeats were the most diverse, comprising five motifs (Fig. 3B). All motifs were predominantly composed of A/T sequences, constituting 66.76–70.13% of the total SSRs.

**Fig. 4:**
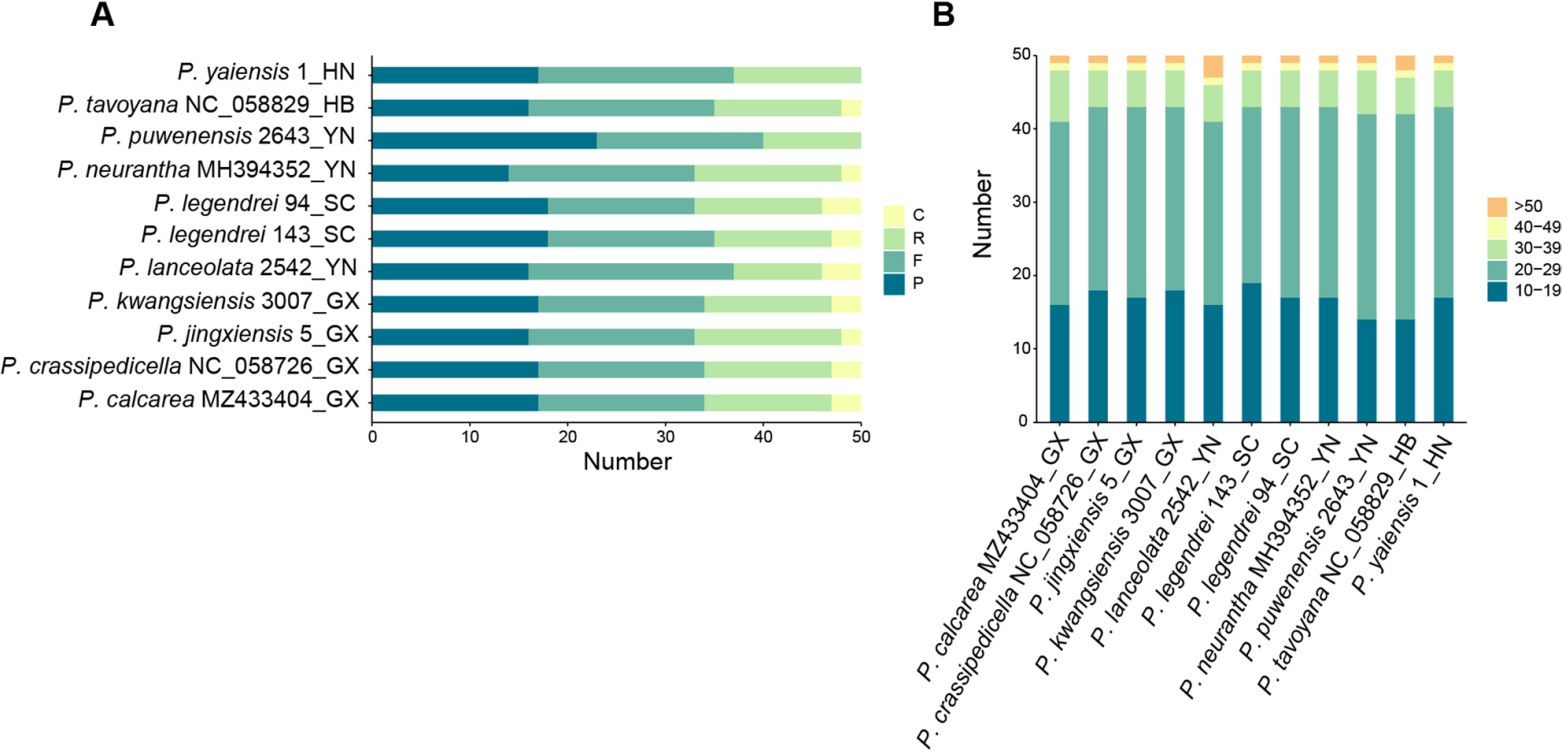
Statistics of dispersed repeats in 11 plastid genomes. **A**: Number of the four dispersed repeats; **B**: Size distribution of dispersed repeats. The ending two capitalized letters of the sequence name denote the initials of the collection province

Fifty dispersed repeats were identified in each of the 11 sequences using REPuter, but there were significant differences in the number of F, P, R, and C repeats. F, P, and R repeats were more common than C repeats (Fig. 4A). The lengths of dispersed repeats clustered between 10 bp and 39 bp, with the most common length being 20–29 bp (24–28), and the least common length being > 40 bp (2–4) (Fig. 4B).

#### Plastome comparison and identification of variation hotspots

**Fig. 5:**
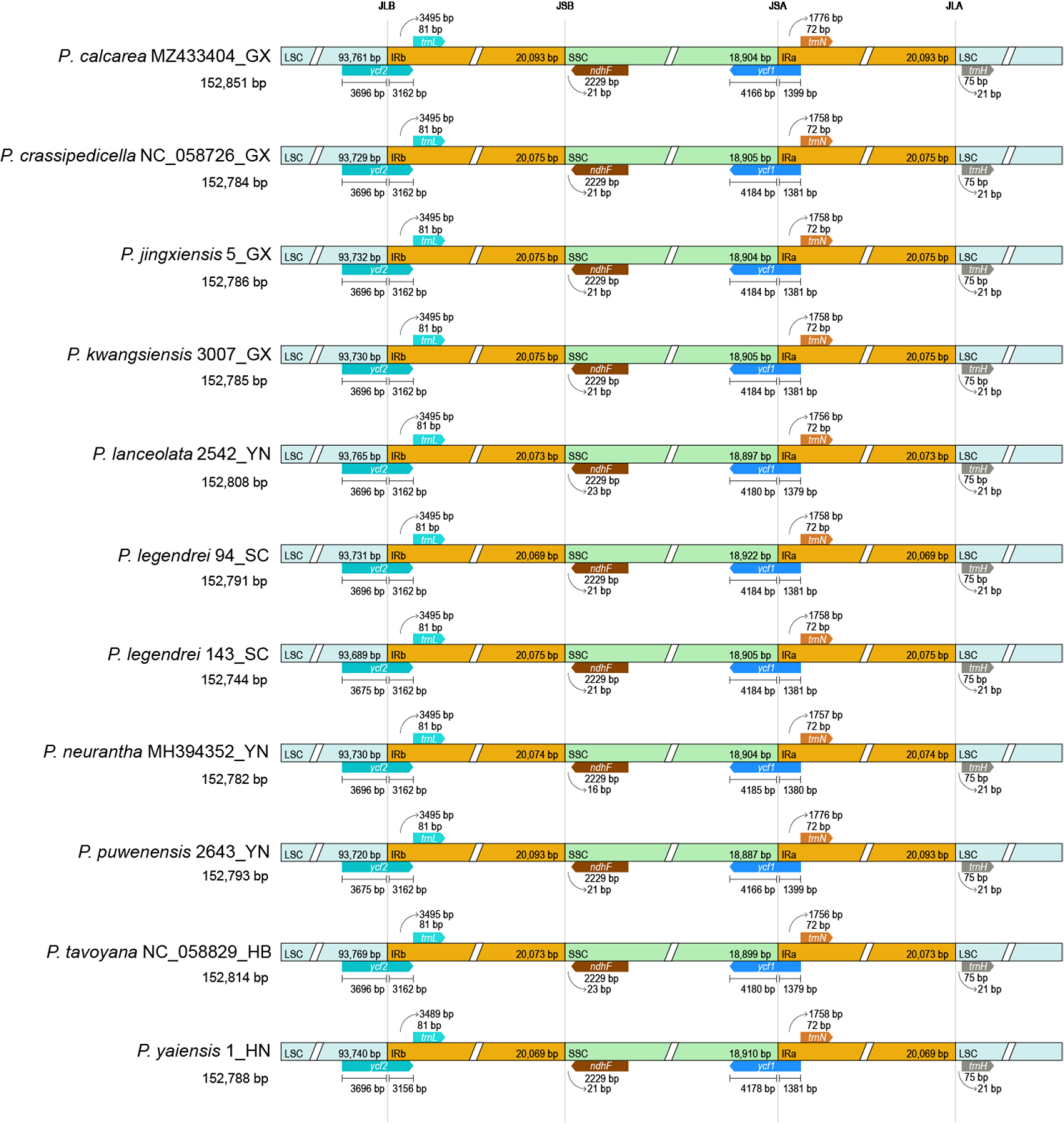
IR boundary analysis of 11 plastomes in the genus *Phoebe*. The ending two capitalized letters of the sequence name denote the initials of the collection province

The results of IR boundary contraction and expansion analysis showed that the boundaries of the 11 sequences are similar, with consistent gene structures and orders, and no gene rearrangement (Fig. 5). IR boundary contraction and expansion primarily occurred in the SSC/IRa and LSC/IRb regions, which contained complete *ycf1* and *ycf2* pseudogenes. The *ycf1* gene is located at the SSC/IRa boundary, with the *ycf1* gene expanding into the SSC region by 4,166–4,185 bp and into the IRa region by 1,379–1,399 bp. The *ycf2* gene is located at the LSC/IRb boundary, with the *ycf2* gene extending 3,156–3,162 bp into the IRb region and 3,675–3,696 bp into the LSC region.

**Fig. 6:**
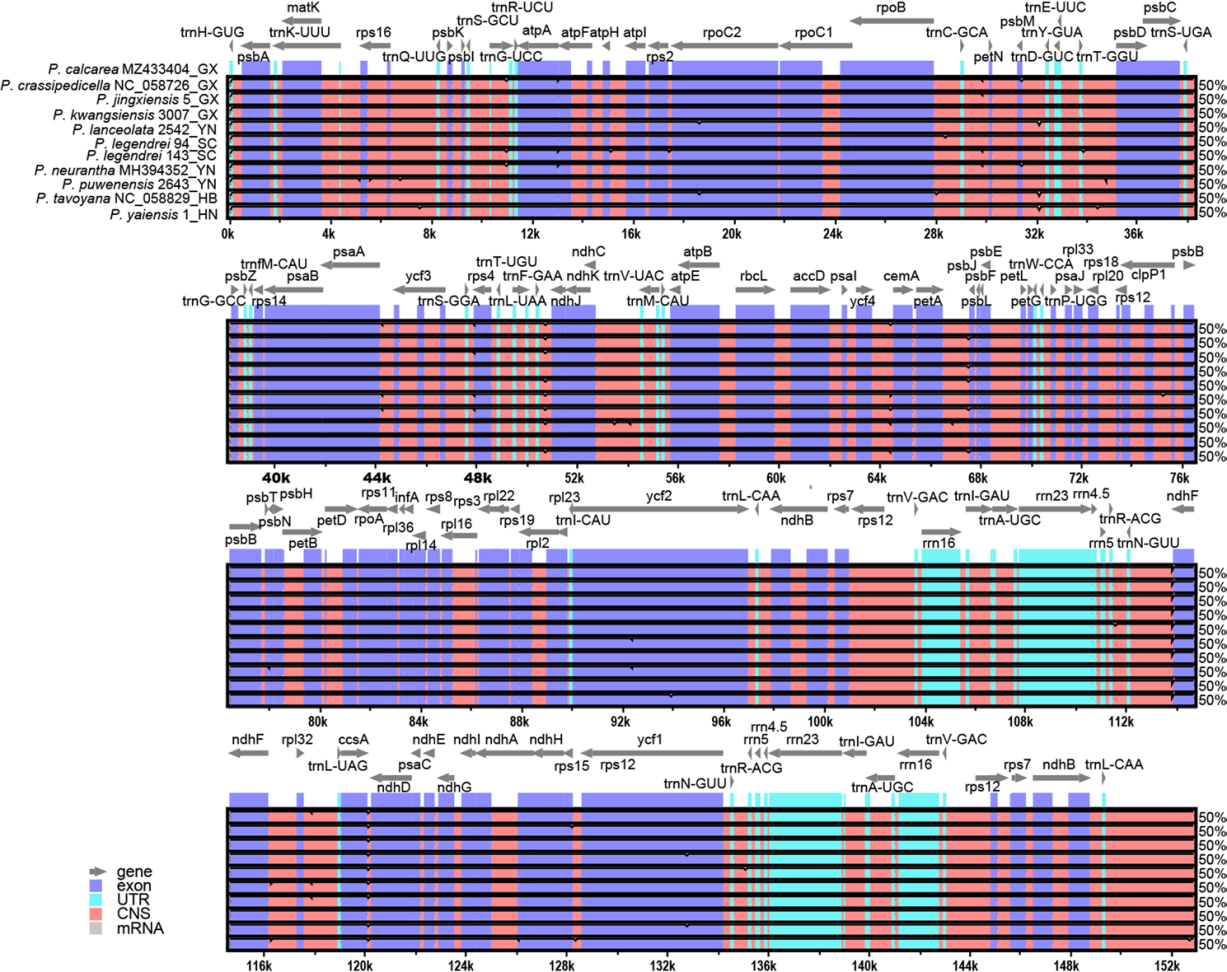
Sequence alignment of 11 plastomes in the genus *Phoebe*. The ending two capitalized letters of the sequence name denote the initials of the collection province

The synteny analysis revealed that the coding regions of the 11 plastomes were more conservative than the non-coding regions, with overall high homogeneity (Fig. 6). A total of 307 variations were identified, they were mainly located in non-coding regions (259 variations consisting of 90 SNPs and 169 indels), compared to coding regions (48 variations comprising 36 SNPs and 12 indels) (Table S3). This indicates that non-coding regions provide more informative sites for phylogenomic analysis.

**Fig. 7:**
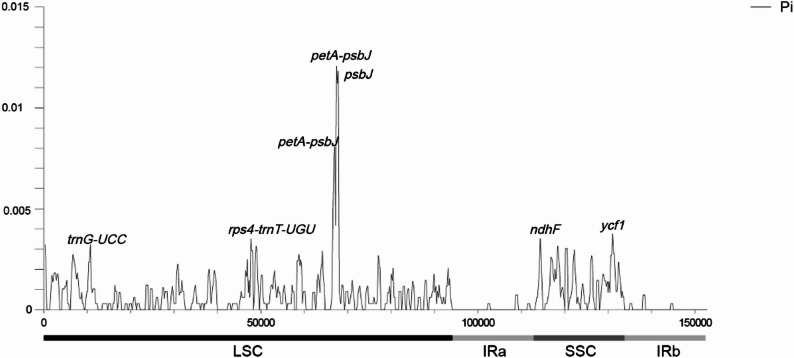
Nucleotide diversity (Pi) of 11 plastomes in the genus *Phoebe*

Nucleotide polymorphism (Pi) analysis showed that the Pi values of the 11 plastomes ranged from 0 to 0.01206, with an average value of 0.00113 (Fig. 7). A total of 66 variable sites (Pi ≥ 0.002) were identified, distributed across the LSC and SSC regions. We identified seven highly variable regions (Pi ≥ 0.004) that are concentrated in the LSC region between the *petA* and *psbL* genes. The region with the highest Pi value is the intergenic region *petA*–*psbJ* in the LSC (0.01206), which can serve as a molecular marker for *Phoebe* species.

#### Phylogenomic analyses

In this study, we reconstructed maximum likelihood (ML) and Bayesian inference (BI) trees using 64 plastomes, with an increased number of samples per species (*N* ≥ 2). The aligned and trimmed data matrix was 152,202 bp long and contained 1,330 bp parsimony informative sites.

**Fig. 8:**
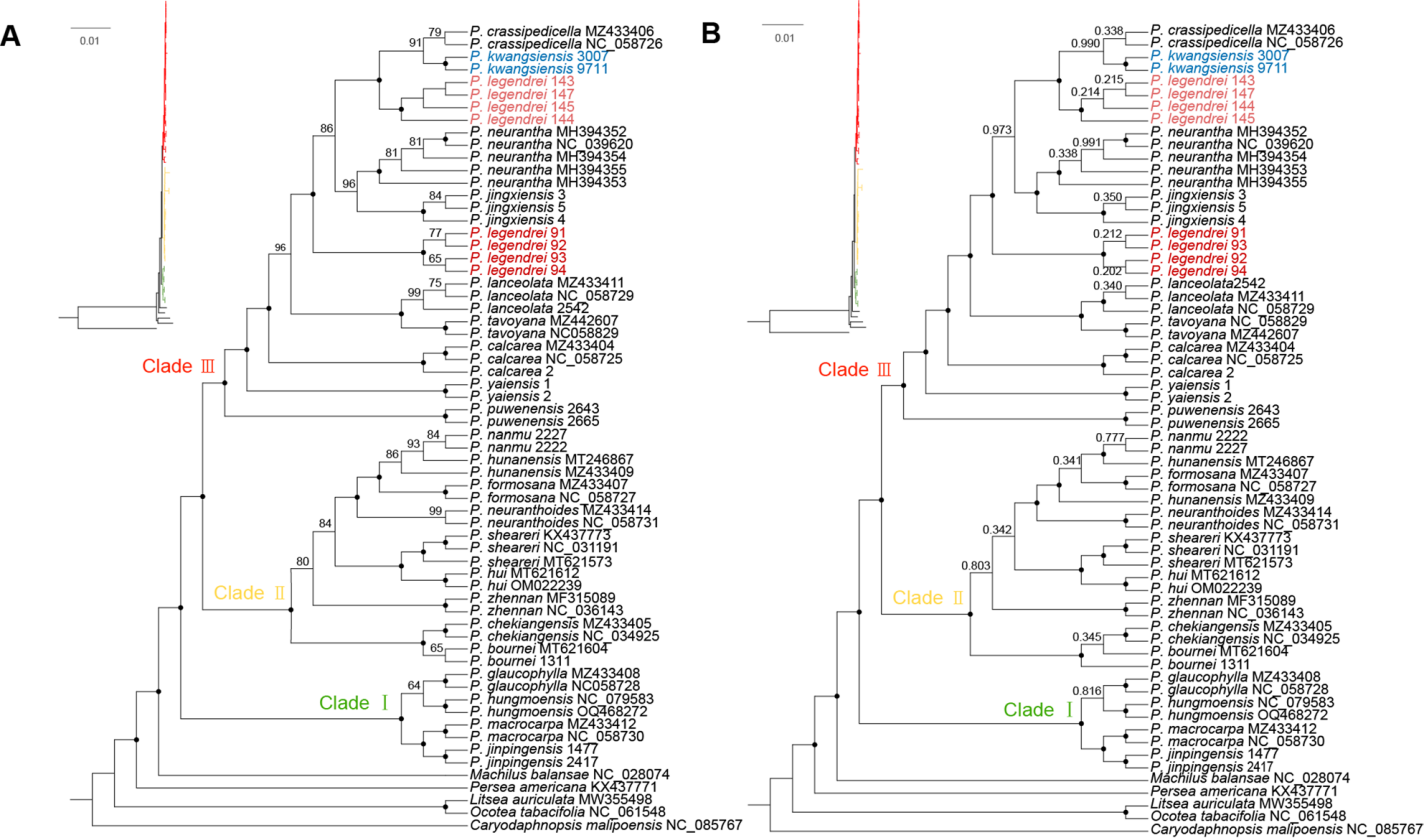
Maximum likelihood (ML) (**A**) and Bayesian inference (BI) (**B**) phylogenomic trees based on 64 plastomes. The ML ultrafast bootstrap (UFboot) and Bayesian posterior probability (BPP) of each node is shown above the branch. A black circle at the node indicates 100% support. The phylogenomic tree with clade lengths is shown in the upper left-hand corner

In the ML and BI trees, the 23 *Phoebe* species are divided into three major clades (UFboot = 100%, BPP = 1) (Fig. 8). Clade I is the earliest diverged clade in the phylogenomic trees and sister to a larger clade including clade II and clade III. Clade II and clade III are sister to one another. The newly sequenced *P. legendrei* and *P. kwangsiensis* are located in clade III. The two samples of *P. kwangsiensis* form a subclade which is sister to *P. crassipedicella* (UFboot = 91%, BPP = 0.99). However, the eight samples of *P. legendrei* are not monophyletic, representing two separate subclades (UFboot = 100%, BPP = 1): *P. legendrei* 143, 144, 145, and 147 form a subclade, with *P. kwangsiensis* and *P. crassipedicella* as its sister group; *P. legendrei* 91, 92, 93, and 94 form another subclade sister to a clade encompassing *P. kwangsiensis* + *P. crassipedicella* + *P. legendrei* 143, 144, 145, and 147 + *P. jingxiensis* + *P. neurantha*.

#### Morphological comparison analyses

**Fig. 9:**
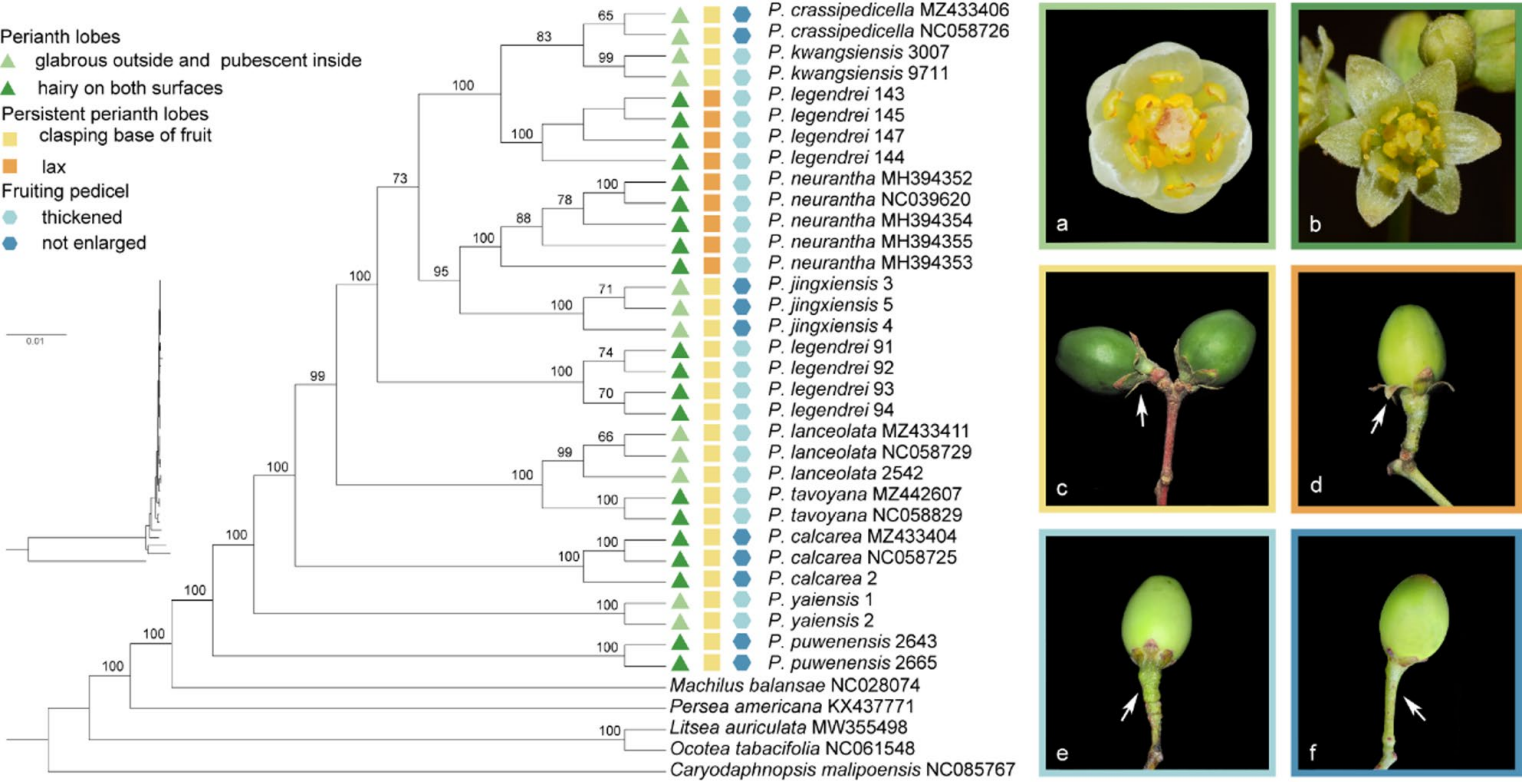
The maximum likelihood (ML) tree of Clade III and the corresponding three groups of morphological characteristics. On the left-hand side is the ML tree of Clade III and the characteristics of the included species, with the UFboot shown at the front of the branch (1–100 %). On the right-hand side are photographs displaying the morphological characters of the three groups, a: *P. jingxiensis* J.Y.Lin et R.H.Jiang(Lin et al., 2024), b–c: *P. legendrei* 91, d:* P. legendrei *143, e: *P. crassipedicella *S.Lee et F.N.Wei, f: *P. kwangsiensis. *Photos by Chengyan Shao, Zhi Yang, and Jianyong Lin

By means of morphological comparison, we found that *P. legendrei* 91, 92, 93, and 94 and *P. legendrei* 143, 144, 145, and 147 differ significantly in terms of leaf shape and size, shape of the perianth lobes, adherence of persistent perianth lobes to fruit, and length of fruiting pedicel (Fig. 9, Table S4). *Phoebe legendrei* 91, 92, 93, and 94 possess elliptic or elliptic-lanceolate leaves, 5.2–12.8 (13–14) long and 1.3–3.8 cm broad, the outer whorl of perianth lobes smaller than the inner whorl, the outer whorl being ovate-triangular, while the inner whorl is ovate-oblong, the persistent perianth lobes often clasping the base of fruit, and fruiting pedicel 0.22–0.63 cm long. The leaves of *P. legendrei* 143, 144, 145, and 147 are lanceolate, oblanceolate, elliptic, or obovate, 7–12.9 long and 2–3.6 cm broad, the perianth lobes ovate-oblong and subequal, persistent perianth lobes often loose, with apex extrorse, and the fruiting pedicel 0.51–0.96 cm long. *Phoebe legendrei* 91, 92, 93, and 94 are morphologically similar to *P. hui*, but differ from the latter by their longer leaves (5.2–12.8 (13–14) cm vs. 5–8 (10) cm), having fewer lateral veins (5–8 pairs vs. 10–12 pairs in the latter), fruit pedicel thicker (thickened vs. not enlarged). Furthermore, *P. legendrei* 143, 144, 145, and 147 are morphologically similar to *P. neurantha*, but differ from the latter by the elevated midrib on the adaxial surface (vs. impressed midrib in the latter species), their ovate-lanceolate and subequal perianth lobes (vs. perianth lobes ovate-oblong, with the outer whorl shorter and narrower, and the inner whorl longer and wider, with a blunt apex), and the thickened fruit pedicel (vs. the fruiting pedicel not enlarged or only slightly thickened in *P. neurantha*).

**Fig. 10:**
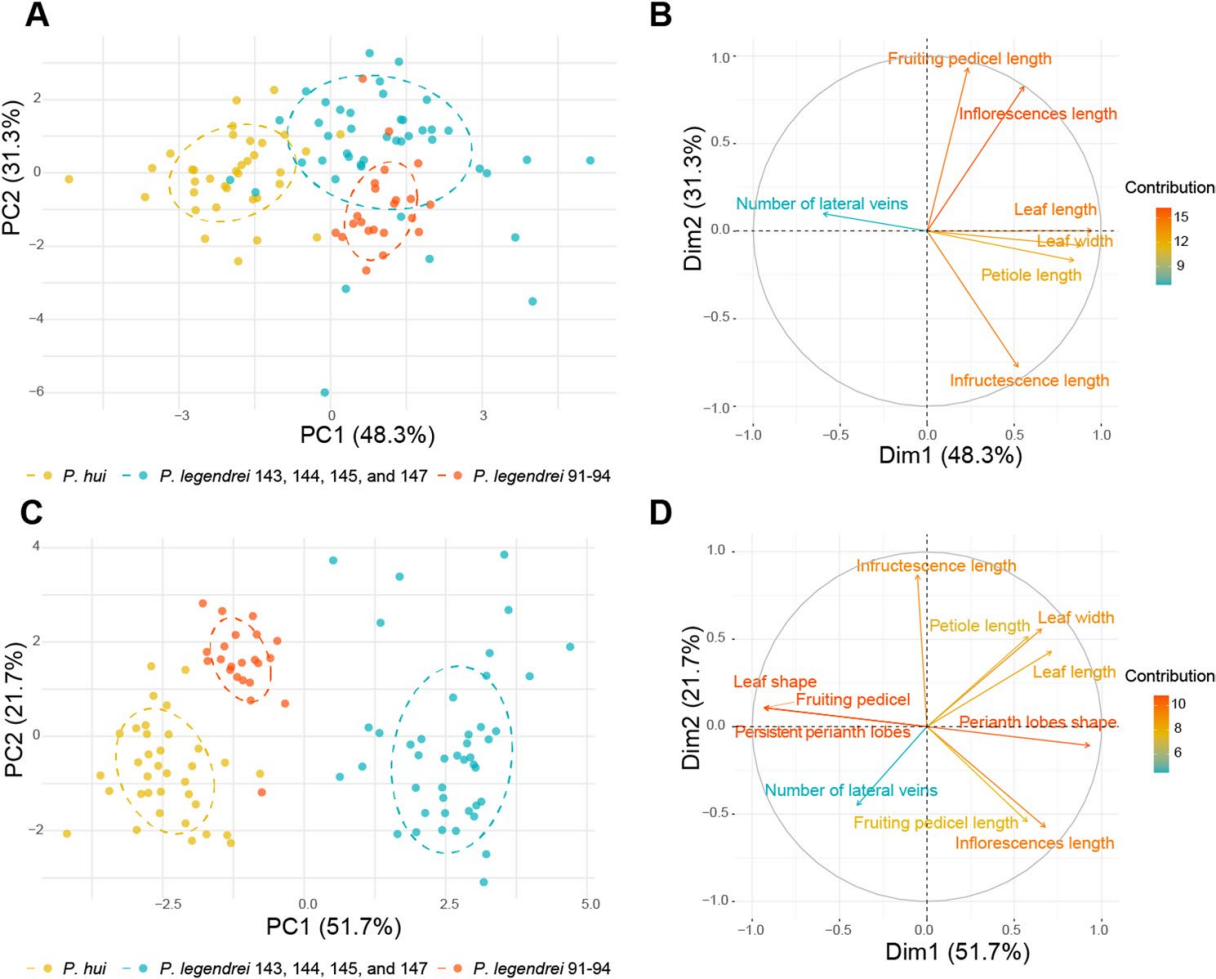
Principal component analyses (PCA) of morphological characters of *Phoebe legendrei* 91–94, *P. legendrei*143, 144, 145, and 147, and *P. hui*. **A** and **B**: PCA result and variable contributions of quantitative characters. **C** and **D**: PCA result and variable contributions of quantitative and qualitative characters

Principal component analysis (PCA) based on quantitative characters revealed differentiation among the three species (Table S5, Fig. 10A). PC1 accounted for 48.3% of the variance, and leaf length, leaf width, and petiole length were the primary contributors (Fig. 10B). While the variation in PC2 (31.3%) was mainly caused by inflorescence length, infructescence length, and fruiting pedicel length (Fig. 10B). The three species were clearly differentiated in both quantitative and qualitative characters (Table S5, Fig. 10C), with PC1 and PC2 explaining 51.7% and 21.7% of the variance, respectively. Variation in PC1 was largely contributed by leaf shape, perianth lobes shape, fruiting pedicel (thickened or not enlarged), and persistent perianth lobes (loose or clasping the fruit) (Fig. 10D), whereas PC2 was mainly influenced by leaf width, inflorescences length, infructescence length, and fruiting pedicel length (Fig. 10D). Thus, the two clades of *P. legendrei* differ from closely related species in their morphology.

## Discussion

In this study, we report the complete plastomes of *P. legendrei* and *P. kwangsiensis* for the first time. We confirm that the genus possesses rather conservative plastome structures [3, 11]. We also reconstruct phylogenomic trees with the hitherto largest dataset of plastomes of the genus *Phoebe*. Our result shows that the genus *Phoebe* contains three major clades (I, II, III), which is consistent with previous phylogenomic studies based on plastomes [3, 10, 11]. Our newly sequenced species (*P. legendrei* and *P. kwangsiensis*) belong to clade III. In clade III, *P. kwangsiensis* is sister to *P. crassipedicella*, but the samples of *P. legendrei* do not comprise a single clade but form two separate subclades. *Phoebe legendrei* 143, 144, 145, and 147 form a subclade sister to *P. kwangsiensis* and *P. crassipedicella*, while *P. legendrei* 91, 92, 93, and 94 form a subclade sister to a large clade including *P. kwangsiensis*, *P. crassipedicella*, *P. legendrei* 143, 144, 145, and 147, *P. jingxiensis*, and *P. neurantha*.

Although the plastomes of *Phoebe* species are highly conservative [11, 13, 19], the sequences of *P. legendrei* 94 and *P. legendrei* 143 are markedly different. In the analysis of dispersed repeats, the number of F, R, C and 10–29 bp repeats are different in *P. legendrei* 94 and *P. legendrei* 143. The IR boundary analysis shows that the *ycf2* gene extends to the LSC region by 3,696 bp and 3,675 bp in the plastomes of *P. legendrei* 94 and *P. legendrei* 143, respectively. Moreover, we found that *P. legendrei* 94 has only 6 highly variable regions, while *P. legendrei* 143 possesses 21 highly variable regions.

Our morphological studies confirm the phylogenomic finding. *Phoebe legendrei* 91, 92, 93, and 94 and *P. legendrei* 143, 144, 145, and 147 are different in leaf shape (elliptic or elliptic-lanceolate vs. lanceolate, oblanceolate, elliptic or obovate), shape of the perianth lobes (outer lobes ovate-triangular, inner lobes ovate-oblong vs. all lobes ovate-oblong), length of fruiting pedicel (0.22–0.63 cm vs. 0.51–0.96 cm) and persistent perianth lobes (clasping the base of fruit vs. loose). This distinction is also corroborated by our PCA result of 11 quantitative and qualitative characters (Table S5, Fig. 10C).

Finally, we examined the protologue, type specimens (Holotype: China. Sichuan, Yalong River, alt. 2000 m, Apr. 30th, 1911, *A.F. Legendre 817* (P00752530)) and other specimens of *P. legendrei*. We found that the morphological characters of *P. legendrei* 143, 144, 145 and 147 are consistent with the morphological description of the protologue [58]. However, the morphology of the remaining four samples (*P. legendrei* 91, 92, 93, and 94) is distinctly different from the type specimens of *P. legendrei*, and related species (Table S4). As a result, we propose that *P. legendrei* 91, 92, 93, and 94 represent a new species of *Phoebe*, which we describe as follows.

### Taxonomic treatment

**Fig. 11:**
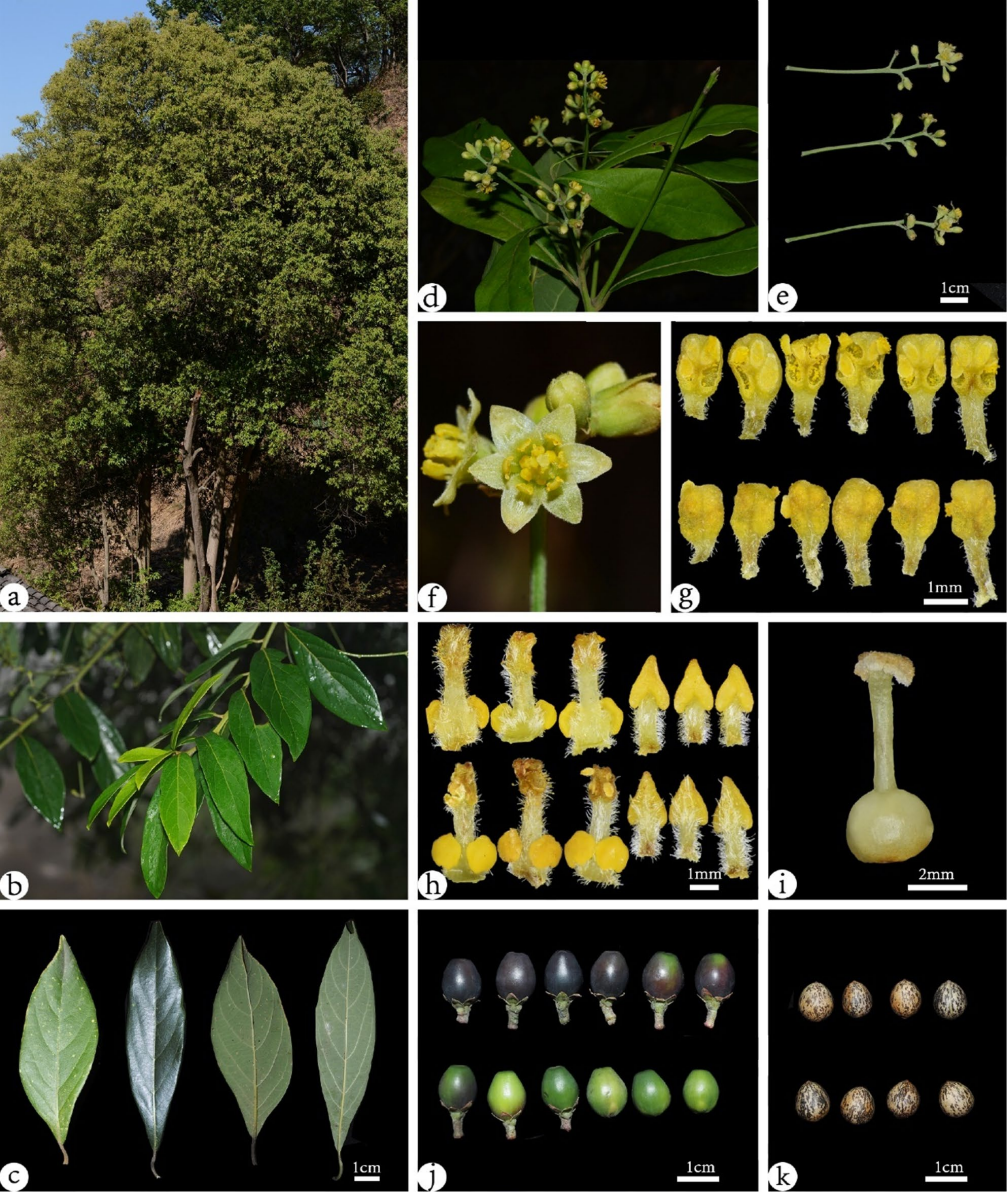
Morphological characters of *P. panzhihuaensis* sp. nov..**a**: Group of trees; **b**: Branch; **c**: Adaxial and abaxial leaf surfaces;**d**: Flowering branch; e: Inflorescences; **f**: Flowers (male phase); **g**: Adaxial and abaxial fertile stamens of the 1st and 2nd whorls; **h**: Adaxial and abaxial fertile stamens of the 3rd whorl and staminodes;**i**: Pistil; **j**: Fruits; **k**: Seeds. Photos by Zhi Yang, Chengyan Shao, Jiayi Song, Yong Yang

*Phoebe panzhihuaensis* Zhi Yang, C.Y. Shao et Y. Yang, sp. nov. (Fig. 11).

Type CHINA. Sichuan Province: Panzhihua City, Panzhihua Cycad National Nature Reserve, Zhulinpo, 26°38′43.70″N, 101°31′35.20″E, elev. 2,092 m, 6 May 2023. Z. Yang, Y.Y. Ye, X.T. Zhang, C.Y. Shao, Y. Sun, PAN05-004 (Holotype: NF; Isotypes: NF).

Diagnosis The new species is similar to *P. legendrei* and *P. hui* in morphology, but differs from *P. legendrei* by its outer perianth lobes being smaller than the inner ones (vs. subequal), fruiting pedicel shorter (0.22–0.63 cm vs. 0.51–0.96 cm), persistent perianth lobes usually clasping base of fruit (vs. lax). It differs from *P. hui* in its longer leaves (5.2–12.8 (13–14) cm vs. 5–8 (10) cm), fewer lateral veins (5–8 vs. 10–12 pairs), and thicker fruiting pedicel (distinctly thickened vs. not enlarged).

Description Evergreen trees, 9–19 m tall. Branchlets initially densely brown pubescent, afterwards glabrate. Leaf blade elliptic or elliptic-lanceolate, 5.2–12.8 (13–14) × 1.3–3.8 cm, leathery, apex acuminate, base cuneate, adaxially glabrous, abaxially sparsely hairy when young, later glabrous, midrib raised on both surfaces, lateral veins 5–8 pairs, abaxially conspicuous, brochidodromous; petiole 0.6–1.7 cm, pubescent, later glabrate. Inflorescences 3.5–9 (10) cm, pubescent. Outer perianth lobes slightly shorter, ovate-triangular, inner lobes ovate-oblong, densely villous on both surfaces; filaments villous, those of 3rd series with subsessile glands at base, staminodes triangular with villous stalks. Ovary globose; style straight, glabrous; stigma capitate. Infructescences 5.4–11.5 (12) cm, pubescent. Fruit broad-ovoid or ellipsoidal, 0.9–1.2 × 0.8–1 cm, apex usually distinctly truncate; fruiting pedicel 0.22–0.63 cm, distinctly thickened; persistent perianth lobes usually clasping base of fruit. Seeds broadly ovoid, surface striated.

Etymology The epithet ‘*panzhihuaensis*’ refers to the type locality, Panzhihua City (Sichuan, China).

Phenology Flowering in May; fruiting in September.

Distribution and habitat So far, *Phoebe panzhihuaensis* has only been found in the dry-hot valley of Panzhihua City.

Conservation status *Phoebe panzhihuaensis* is assessed as Critically Endangered (CR) under the IUCN Red List Categories and Criteria [59], with approximately 10 mature individuals and an extremely restricted distribution.

Paratypes CHINA. Sichuan Province: Panzhihua City, Panzhihua Cycad National Nature Reserve, Zhulinpo, 26°38′58.13″N, 101°31′30.16″E, elev. 2,062 m, 9 Sept. 2024, J.B. Lin, C01-C and C02-B (NF); 28 June 2022, Y. Yang, Z. Yang, L.L. Wang, C. Tan, Z.X. Yu, PAN02-092 (NF).

### Key to *Phoebe panzhihuaensis* and closely related species of the genus *Phoebe*

1. Perianth lobes glabrous or puberulent outside, hairy inside.

 2 . Fruiting pedicel thickened··································································*P. crassipedicella*.

 2. Fruiting pedicel not enlarged.

 3. Branchlets and leaf blade glabrous···························································*P. jingxiensis*.

 3. Branchlets pubescent; leaf blade adaxially glabrous or hairy along midrib, abaxially gray-brown pubescent····················································································*P. kwangsiensis*.

1. Perianth lobes pubescent on both surfaces.

 4. Leaf midrib impressed adaxially; fruiting pedicel not enlarged or only slightly thickened.

 5. Leaves 8–16 cm long, abaxially pubescent, glabrescent; persistent perianth lobes not clasping the base of the ovoid fruits·········································································*P. neurantha*.

 5. Leaves 5–8 (10) cm long, abaxially densely pubescent; persistent perianth lobes clasping the base of the ellipsoid fruits····················································································*P. hui*.

4. Leaf midrib elevated adaxially; fruiting pedicel distinctly thickened.

 6. Perianth lobes subequal, not clasping the base of fruits; fruiting pedicel 0.51–0.96 cm long································································································*P. legendrei*.

 6. Perianth lobes unequal, usually clasping the base of fruits; fruiting pedicel 0.22–0.63 cm long························································································*P. panzhihuaensis*.

## Conclusions

In this study, a new plastome phylogenomic and taxonomic study of *P. legendrei* and closely related species was conducted. We sequenced the plastomes of two additional species (*P. legendrei* and *P. kwangsiensis*) that had not been reported in previous studies, and reconstructed phylogenomic trees containing the largest dataset of plastomes of the genus *Phoebe* until now. The reconstructed phylogenomic trees of *Phoebe* contains three strongly supported clades. By integrating phylogenomic and morphological evidence, we resolved the species delimitations of *P. legendrei* and its closely related species including *P. kwangsiensis*, *P. crassipedicella*, and *P. neurantha*. In addition, our phylogenomic and morphological results consistently supported the proposal of a new species, i.e., *Phoebe panzhihuaensis* sp. nov. Our findings lay a solid foundation for both conservation and sustainable utilization of *Phoebe* in East Asia. However, our molecular phylogenomic trees are based solely on plastome data, and further nuclear genomic data will be essential to confirm the results.

## Supplementary Information

Supplementary Material 1: Characteristics of 11 plastomes in the genus *Phoebe.*

Supplementary Material 2: Statistics of the Ka/Ks values for protein-coding genes.

Supplementary Material 3: Variation types and counts in both coding and non-coding regions across the 11 plastomes.

Supplementary Material 4: Comparison of morphological characters of *P. legendrei* and five related species.

Supplementary Material 5: Quantitative and qualitative characters obtained from 100 specimens.

## Data Availability

All newly sequenced plastomes have been submitted to the National Center for Biotechnology Information (NCBI).

## References

[1] Nees CG. Systema Laurinarum. Berolini: Sumptibus Veitii et Sociorum; 1836. p. VIII+720.

[2] Li L, Liu B, Song Y, Meng HH, Ci XQ, Conran JG, et al. Global advances in phylogeny, taxonomy and biogeography of Lauraceae. Plant Divers. 2025;47:341–64. 40496996 10.1016/j.pld.2025.04.001 PMC12146874

[3] Song Y, Yao X, Tan YH, Gan Y, Yang JB, Corlett RT. Comparative analysis of complete chloroplast genome sequences of two subtropical trees, *Phoebe Sheareri* and *Phoebe omeiensis* (Lauraceae). Tree Genet Genomes. 2017;13:120.

[4] Li HW, Li J, Huang PH, Wei FN, Cui HB, van der Werff H. Lauraceae. In: Wu ZY, Raven PH, Hong DY, editors. Flora of China. Volume 7. Beijing & Saint Louis: Science Press & Missouri Botanical Garden; 2008. pp. 102–254.

[5] Li HW, Pai PY, Li YR, Lee SK, Wei FN, Wei YZ, et al. Lauraceae. In: Delectis Florae Reipublicae Popularis Sinicae Agendae Academiae Sinicae Edita editor. Flora reipublicae popularis sinicae. Volume 31. Beijing: Science Press; 1982;1–463.

[6] Li L, Li J, Li HW. Taxonomic revision of five species of the genus *Phoebe* (Lauraceae) from China. Plant Divers Resour. 2011;33:157–60.

[7] Li L, Li J, Rohwer JG, van der Werff H, Wang ZH, Li HW. Molecular phylogenetic analysis of the *Persea* group (Lauraceae) and its biogeographic implications on the evolution of tropical and subtropical amphi-Pacific disjunctions. Am J Bot. 2011;98:1520–36. 21860056 10.3732/ajb.1100006

[8] Mo YQ, Li L, Li JW, Rohwer JG, Li HW, Li J. *Alseodaphnopsis*: a new genus of Lauraceae based on molecular and morphological evidence. PLoS ONE. 2017;12:e0186545. 29045488 10.1371/journal.pone.0186545 PMC5646853

[9] Liu ZF, Ma H, Ci XQ, Li L, Song Y, Liu B, et al. Can plastid genome sequencing be used for species identification in lauraceae? Bot J Linn Soc. 2021;197:1–14.

[10] Xiao TW, Yan HF, Ge XJ. Plastid phylogenomics of tribe Perseeae (Lauraceae) yields insights into the evolution of East Asian subtropical evergreen broad-leaved forests. BMC Plant Biol. 2022;22:32. 35027008 10.1186/s12870-021-03413-8 PMC8756638

[11] Shi WB, Song WC, Chen ZM, Cai HH, Gong Q, Liu J, et al. Comparative chloroplast genome analyses of diverse *Phoebe* (Lauraceae) species endemic to China provide insight into their phylogeographical origin. PeerJ. 2023;11:e14573. 36755871 10.7717/peerj.14573 PMC9901306

[12] Lin JY, Jiang DD, Ou HB, Li J, He YH, Jiang RH. *Phoebe jingxiensis* (Lauraceae), a new species from Guangxi, China. Phytotaxa. 2024;664:207–13.

[13] Li YG, Xu WQ, Zou WT, Jiang DY, Liu XH. Complete chloroplast genome sequences of two endangered *Phoebe* (Lauraceae) species. Bot Stud. 2017;58:37. 28905330 10.1186/s40529-017-0192-8 PMC5597560

[14] Semwal DK, Semwal RB. Ethnobotany, Pharmacology and phytochemistry of the genus *Phoebe* (Lauraceae). Mini-Rev Org Chem. 2013;10:12–26.

[15] Zhang H, Chase JM, Liao J. Habitat amount modulates biodiversity responses to fragmentation. Nat Ecol Evol. 2024;8:1437–47. 38914711 10.1038/s41559-024-02445-1

[16] Ministry of Ecology and Environment. Redlist of China’s Biodiversity—Higher Plants. (2020). 2023. https://www.mee.gov.cn/xxgk2018/xxgk/xxgk01/202305/t20230522_1030745.html. Accessed 15 Mar 2025.

[17] National Forestry and Grassland Administration and Ministry of Agriculture and Rural Affairs. National Key Protected Wild Plants of China. 2021. https://www.gov.cn/zhengce/zhengceku/2021-09/09/content_5636409.htm. Accessed 15 Mar 2025.

[18] Shang CB. A new species of the *Phoebe* from Chekiang. Acta Phytotax Sin. 1974;12:295.

[19] Song Y, Yu WB, Tan YH, Jiu JJ, Wang B, Yang JB, et al. Plastid phylogenomics improve phylogenetic resolution in the Lauraceae. J Syst Evol. 2020;58:423–39.

[20] Zou HY, Wu DR. Taxonomic notes of *Phoebe bournei*. In: Zou HY, Wu DR editors. Population Ecology of Bourne *Phoebe* (*Phoebe bournei*). Beijing: China Forestry Publishing Press; 1997. p. 2–4.

[21] Ding X, Xiao JH, Li L, Conran JG, Li J. Congruent species delimitation of two controversial gold-thread Nanmu tree species based on morphological and restriction site-associated DNA sequencing data. J Syst Evol. 2019;57:234–46.

[22] Wei FN. Materials of genus *Phoebe* Nees from China. Guihaia. 1983;3:7–10.

[23] Karbstein K, Kösters L, Hodač L, Hofmann M, Hörandl E, Tomasello S, et al. Species delimitation 4.0: integrative taxonomy Meets artificial intelligence. Trends Ecol Evol. 2024;39:771–84. 38849221 10.1016/j.tree.2023.11.002

[24] Zhang Q, Sun TT, Omollo WO, Le TC, Nguyen VH, Chen ZD, et al. An integrative taxonomy of Asian *Caryodaphnopsis* (Lauraceae) based on morphology and phylogenomics. Taxon. 2024;73:949–70.

[25] Huang L, Yang YP, Liu XY, Qiu LF, Li YY, Ma ZW, et al. Integrated analyses reveal extensive cytonuclear discordance and two new members of *Rhodiola*. J Syst Evol. 2025;63:737–52.

[26] van Elst T, Sgarlata GM, Schüßler D, Tiley GP, Poelstra JW, Scheumann M, et al. Integrative taxonomy clarifies the evolution of a cryptic primate clade. Nat Ecol Evol. 2025;9:57–72. 39333396 10.1038/s41559-024-02547-w PMC11726463

[27] Yuan YM, Feng Y, Wang JB, Ulah F, Yuan M, Gao YD. Integrative taxonomy for species delimitation: a case study in two widely accepted yet morphologically confounding *Rosa* species within sect. *Pimpinellifoliae* (Rosaceae). Mol Ecol. 2025;e17779. 10.1111/mec.17779 10.1111/mec.17779 40285506

[28] Peng HW, Wang W. Phylogenetic tree reconstruction based on molecular data. Chin Bull Bot. 2023;58:261–73.

[29] Li JL, Wang S, Yu J, Wang L, Zhou SL. A modified CTAB protocol for plant DNA extraction. Chin Bull Bot. 2013;48:72–8.

[30] Jin JJ, Yu WB, Yang JB, Song Y, dePamphilis CW, Yi TS, et al. GetOrganelle: a fast and versatile toolkit for accurate de novo assembly of organelle genomes. Genome Biol. 2020;21:241. 32912315 10.1186/s13059-020-02154-5 PMC7488116

[31] Wick RR, Schultz MB, Zobel J, Holt KE. Bandage: interactive visualisation of de novo genome assemblies. Bioinformatics. 2015;31(20):3350–2. 26099265 10.1093/bioinformatics/btv383 PMC4595904

[32] Tillich M, Lehwark P, Pellizzer T, Ulbricht-Jones ES, Fischer A, Bock R, et al. GeSeq- versatile and accurate annotation of organelle genomes. Nucleic Acids Res. 2017;45:W6–11. 28486635 10.1093/nar/gkx391 PMC5570176

[33] Shi L, Chen H, Jiang M, Wang L, Wu X, Huang L, et al. CPGAVAS2, an integrated plastome sequence annotator and analyzer. Nucleic Acids Res. 2019;47:W65–73. 31066451 10.1093/nar/gkz345 PMC6602467

[34] Kearse M, Moir R, Wilson A, Stones-Havas S, Cheung M, Sturrock S, et al. Geneious basic: an integrated and extendable desktop software platform for the organization and analysis of sequence data. Bioinformatics. 2012;28:1647–9. 22543367 10.1093/bioinformatics/bts199 PMC3371832

[35] Greiner S, Lehwark P, Bock R. OrganellarGenomeDRAW (OGDRAW) version 1.3.1: expanded toolkit for the graphical visualization of organellar genomes. Nucleic Acids Res. 2019;47:W59–64. 30949694 10.1093/nar/gkz238 PMC6602502

[36] Xiong S, Zhou FQ, Wang SD, Li R, Wang SB, Huang Y. Analysis of chloroplast genomic characteristics and phylogeny in *Sycopsis triplinervia*. Guihaia. 2025;45:855–71.

[37] Tamura K, Stecher G, Kumar S. MEGA11: molecular evolutionary genetics analysis version 11. Mol Biol Evol. 2021;38:3022–7. 33892491 10.1093/molbev/msab120 PMC8233496

[38] Kumar S, Stecher G, Tamura K. MEGA7: molecular evolutionary genetics analysis version 7.0 for bigger datasets. Mol Biol Evol. 2016;33:1870–4. 27004904 10.1093/molbev/msw054 PMC8210823

[39] Xu SJ, Teng K, Zhang H, Gao K, Wu JY, Duan LS, et al. Chloroplast genomes of four *Carex* species: long repetitive sequences trigger dramatic changes in chloroplast genome structure. Front Plant Sci. 2023;14:1100876. 36778700 10.3389/fpls.2023.1100876 PMC9911286

[40] Zhang Z. KaKs_Calculator 3.0: calculating selective pressure on coding and non-coding sequences. Genom Proteom Bioinf. 2022;20:536–40. 10.1016/j.gpb.2021.12.002 PMC9801026 34990803

[41] Hurst LD. The Ka/Ks ratio: diagnosing the form of sequence evolution. Trends Genet. 2002;18:486–7. 12175810 10.1016/s0168-9525(02)02722-1

[42] Beier S, Thiel T, Münch T, Scholz U, Mascher M. MISA-web: a web server for microsatellite prediction. Bioinformatics. 2017;33:2583–5. 28398459 10.1093/bioinformatics/btx198 PMC5870701

[43] Thiel T, Michalek W, Varshney RK, Graner A. Exploiting EST databases for the development and characterization of gene-derived SSR-markers in barley (*Hordeum vulgare* L). Theor Appl Genet. 2003;106:411–22. 12589540 10.1007/s00122-002-1031-0

[44] Kurtz S, Choudhuri JV, Ohlebusch E, Schleiermacher C, Stoye J, Giegerich R. REPuter: the manifold applications of repeat analysis on a genomic scale. Nucleic Acids Res. 2001;29:4633–42. 11713313 10.1093/nar/29.22.4633 PMC92531

[45] Wickham H. Ggplot2: elegant graphics for data analysis. New York: Springer-; 2016.

[46] Li H, Guo Q, Xu L, Gao H, Liu L, Zhou X. CPJSdraw: analysis and visualization of junction sites of chloroplast genomes. PeerJ. 2023;11:e15326. 37193025 10.7717/peerj.15326 PMC10182761

[47] Frazer KA, Pachter L, Poliakov A, Rubin EM, Dubchak I. VISTA: computational tools for comparative genomics. Nucleic Acids Res. 2004;32:W273–9. 15215394 10.1093/nar/gkh458 PMC441596

[48] Rozas J, Ferrer-Mata A, Sánchez-DelBarrio JC, Guirao-Rico S, Librado P, Ramos-Onsins SE, et al. DnaSP 6: DNA sequence polymorphism analysis of large data sets. Mol Biol Evol. 2017;34:3299–302. 29029172 10.1093/molbev/msx248

[49] Song Y, Dong WP, Liu B, Xu C, Yao X, Gao J, et al. Comparative analysis of complete Chloroplast genome sequences of two tropical trees *Machilus yunnanensis* and *Machilus balansae* in the family Lauraceae. Front Plant Sci. 2015;6:662. 26379689 10.3389/fpls.2015.00662 PMC4548089

[50] Katoh K, Standley DM. MAFFT multiple sequence alignment software version 7: improvements in performance and usability. Mol Biol Evol. 2013;30:772–80. 23329690 10.1093/molbev/mst010 PMC3603318

[51] Capella-Gutierrez S, Silla-Martinez JM, Gabaldon T. TrimAl: a tool for automated alignment trimming in large-scale phylogenetic analyses. Bioinformatics. 2009;25:1972–3. 19505945 10.1093/bioinformatics/btp348 PMC2712344

[52] Zhang D, Gao FL, Jakovlić I, Zou H, Zhang J, Li WX, et al. PhyloSuite: an integrated and scalable desktop platform for streamlined molecular sequence data management and evolutionary phylogenetics studies. Mol Ecol Resour. 2020;20:348–55. 31599058 10.1111/1755-0998.13096

[53] Nguyen LT, Schmidt HA, von Haeseler A, Minh BQ. IQ-TREE: a fast and effective stochastic algorithm for estimating maximum-likelihood phylogenies. Mol Biol Evol. 2015;32:268–74. 25371430 10.1093/molbev/msu300 PMC4271533

[54] Minh BQ, Nguyen MA, von Haeseler A. Ultrafast approximation for phylogenetic bootstrap. Mol Biol Evol. 2013;30:1188–95. 23418397 10.1093/molbev/mst024 PMC3670741

[55] Ronquist F, Teslenko M, van der Mark P, Ayres DL, Darling A, Höhna S, et al. MrBayes 3.2: efficient bayesian phylogenetic inference and model choice across a large model space. Syst Biol. 2012;61:539–42. 22357727 10.1093/sysbio/sys029 PMC3329765

[56] Letunic I, Bork P. Interactive tree of life (iTOL) v6: recent updates to the phylogenetic tree display and annotation tool. Nucleic Acids Res. 2024;52:W78–82. 38613393 10.1093/nar/gkae268 PMC11223838

[57] Josse J, Husson F, missMDA. A package for handling missing values in multivariate data analysis. J Stat Softw. 2016;70:1–31.

[58] Lecomte H. Lauracées de Chine et D’Indo-Chine. Paris: Masson et Cie; 1913. p. 103.

[59] IUCN Standards and Petitions Committee. Guidelines for Using the IUCN red list Categories and Criteria. Version 16. Gland: Cambridge; 2024.

